# Exploratory proof-of-concept study and failure mode analysis of a dual-lumen stent graft for transapical off-pump LVAD implantation

**DOI:** 10.1038/s41598-026-68876-x

**Published:** 2026-09-02

**Authors:** Florian Meissner, Manuela Schön, Michelle Costa Galbas, Hendrik Straky, Johannes Dinkelaker, Martin Czerny, Wolfgang Bothe

**Affiliations:** 1 Department for Cardiac, Thoracic, Vascular Surgery and Aortic Medicine, Medical Center—University of Freiburg, Faculty of Medicine, University of Freiburg, Hugstetter Strasse 55, 79106 Freiburg, Germany; 2 Center for Experimental Models and Transgenic Service, Medical Center—University of Freiburg, Faculty of Medicine, University of Freiburg, Stefan-Meier-Strasse 17, 79104 Freiburg, Germany

**Keywords:** Cardiology, Medical research

## Abstract

Transapical off-pump implantation of left ventricular assist devices (LVADs) requires a separate inflow cannula and outflow graft. This study represents the first exploratory proof-of-concept evaluation of a dual-lumen stent graft designed to redirect blood from the left ventricle across the aortic valve into the ascending aorta while enabling LVAD support. The stent graft was tested in a mock circulatory loop, during wet-lab experiments on explanted porcine hearts, and in an exploratory acute large-animal study. In vitro testing showed no relevant impairment of pump performance. Wet-lab experiments confirmed that transapical positioning is feasible while revealing incomplete device expansion and myocardial tearing during balloon-assisted apical displacement. In vivo, a reinforced prototype could be positioned across the aortic valve, but myocardial compression impaired inflow expansion and LV unloading, while insufficient apical sealing resulted in bleeding and air embolism. Postmortem analysis confirmed correct device positioning without macroscopic valvular injury but identified myocardial tearing. These failure modes highlight the need for alternative design strategies for transapical off-pump LVAD implantation.

## Introduction

Advanced heart failure remains a major clinical challenge, affecting an estimated 6.7 million adults in the United States^1^. Up to 10% of these patients develop advanced disease requiring heart transplantation or durable mechanical circulatory support (MCS)^2^. Centrifugal-flow left ventricular assist devices (LVADs), such as the HeartMate 3 (HM3, Abbott Laboratories, Abbott Park, IL), represent the standard of care following the withdrawal of the HeartWare ventricular assist device (HVAD; Medtronic, Dublin, Ireland) in 2021^3^. Conventional LVAD implantation requires median sternotomy and left ventricular (LV) coring under cardiopulmonary bypass (CPB), contributing to surgical complexity and perioperative complications. Less-invasive implantation strategies, such as anterolateral thoracotomy approaches evaluated in the LATERAL trial^4^, have reduced surgical trauma and perioperative complications. However, these approaches still require separate inflow cannula placement into the LV and outflow graft (OG) anastomosis to the ascending aorta (AAo).

To address these limitations, experimental concepts aim to simplify LVAD implantation by enabling transapical off-pump access and combining the LV inflow cannula and OG in a dual-lumen design. Transventricular outflow configurations that cross the aortic valve (AV) avoid an aortic OG anastomosis, thereby enabling transapical implantation through a single access^5–8^.

Transventricular approaches may be advantageous in patients with severe aortic calcification, porcelain aorta, or extensive atherosclerosis, in whom aortic OG anastomosis may be challenging. They preserve the mediastinum for subsequent cardiac surgery (e.g., heart transplantation, LVAD explantation). Compared with aortic OGs, transvalvular OGs or solid cannulas may provide more coaxial antegrade blood flow within the AAo and avoid typical OG complications such as kinking and external compression.

These advantages, however, depend on outflow alignment and centering within the LV outflow tract, the AV, and the AAo. Permanent device–AV interaction introduces biomechanical and hemodynamic concerns regarding cusp interference, reduced effective orifice area, impaired aortic root washout, and potential effects on intraventricular flow patterns and thrombogenicity (Table 1).

**Table 1:** Comparison of conventional aortic and transvalvular LVAD outflows.

Aspect	Conventional aortic outflow	Transvalvular outflow
**Outflow Graft or Outflow Cannula**		
Route	Extracardiac	Intracardiac
End	End-to-side anastomosis to AAo	Crossing AV
Material	Woven polyester graft	Reinforced woven polyester graft or polymer cannula
Outer diameter	10–12 mm	7–12 mm
Typical length	15.3–19.7 cm^9^	13.5–17.1 cm^10^
**Surgical Approach**		
Thoracic access	Median sternotomy or left anterolateral thoracotomy	Left anterolateral thoracotomy
Aortotomy for OG anastomosis	Required, contraindicated in case of aortic calcifications, porcelain aorta, severe atherosclerosis^11^	Not required
Considerations for follow-up surgery	Dense retrosternal adhesions following median sternotomy	Preservation of mediastinum and avoidance of dense adhesions
**Potential Complications**		
Access-specific	Mediastinitis, sternal dehiscence, excessive bleeding, chest pain	Involuntary rib fracture, lung injury, wound infection, neuralgia
Outflow-specific	OG kinking, external compression, stenosis, secondary thrombosis	Apical stasis, decreased pulsatility, thrombus formation^12^
Anastomosis-specific	Suture failure and bleeding, pseudoaneurysm formation	No aortic OG anastomosis
Aortic valve regurgitation	Secondary to reduced AV opening or chronic AV closure	Secondary to permanent device interaction and impaired mobility
Aortic root stasis and thrombus formation	Driven by reduced AV opening and low transvalvular washout	Driven by impaired mobility and eccentric outflow alignment within AV
**Aortic Hemodynamics**		
Flow entry and alignment	Side-entry with 30–60° angled flow	Direct coaxial antegrade flow
Jet impingement	Opposing AAo wall, depending on anastomotic angle and orientation	Proximal outer curvature of distal AAo wall and aortic arch

AAo, ascending aorta; AV, aortic valve; OG, outflow graft.

For transventricular approaches, it is unknown whether expandable devices, such as stent grafts (SGs), that allow controlled transcatheter deployment, are suitable. Deployment without apical coring needs tissue displacement, which may result in altered LV geometry, myocardial tearing, and impaired apical sealing. In addition, cyclic myocardial contraction may cause SG compression.

We designed a novel dual-lumen SG as an LVAD accessory and explored whether it could enable single-access transapical off-pump implantation without coring. We hypothesized that the SG could be deployed transventricularly and across the AV while maintaining LVAD performance and SG patency. This exploratory proof-of-concept study used a staged preclinical workflow to assess the proposed approach and identify failure modes that limit clinical translation. Rather than demonstrating clinical feasibility, we sought to characterize myocardial deformation and apical sealing as well as device positioning across the AV in vitro, ex vivo, and in vivo.

## Methods

### Design and manufacturing

**Fig. 1:**
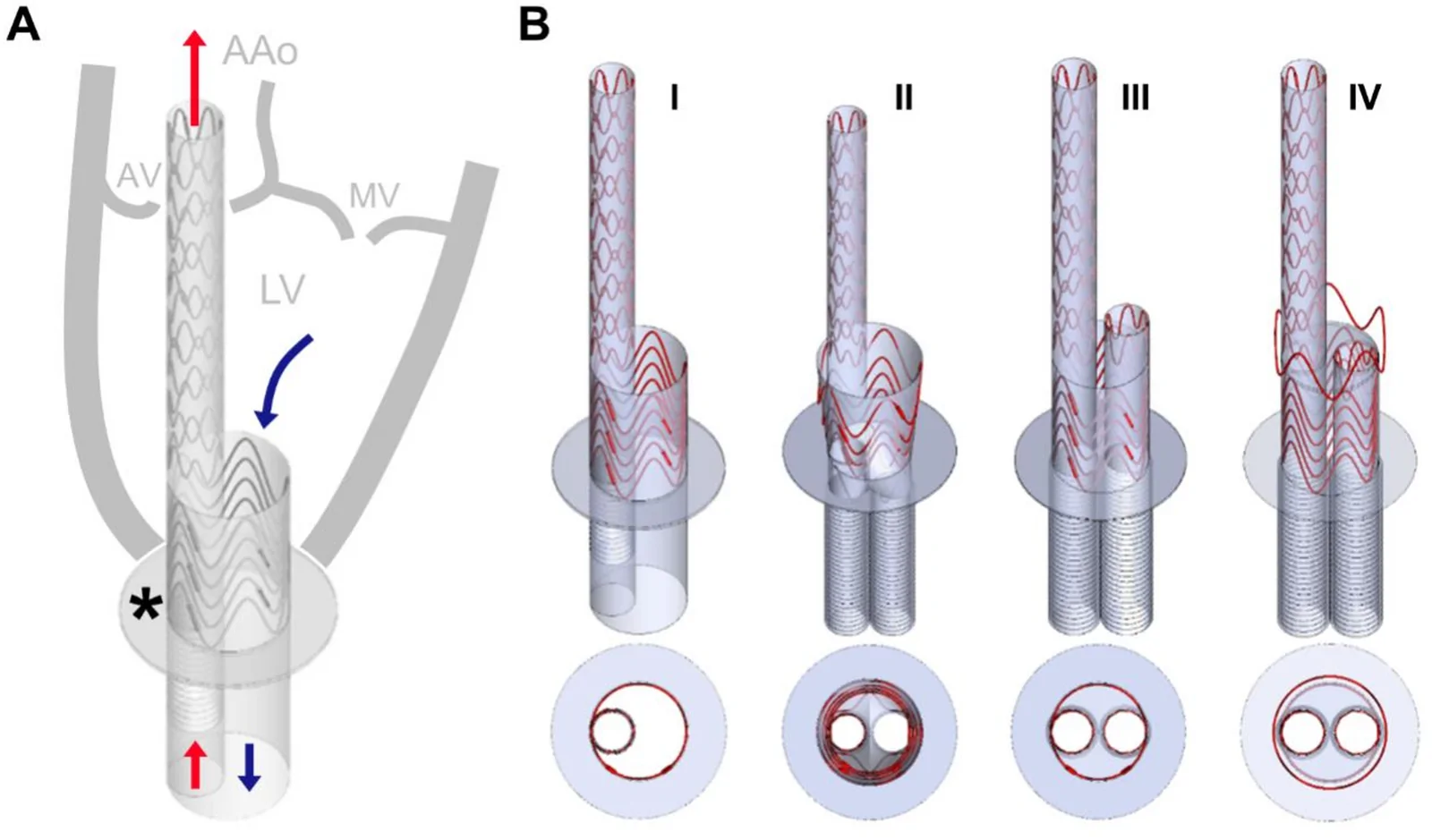
Dual-lumen stent graft for left ventricular (LV) mechanical support. (**A**) Schematic showing the stent graft inside the LV. The inflow (blue arrows) is oriented towards the mitral valve (MV), and the outflow graft (red arrows) crosses the aortic valve (AV), redirecting blood into the ascending aorta (AAo). (**B**) Computer-aided design models of four different stent graft solutions (Concept I–IV). Concepts I and IV were tested in vitro and in vivo, respectively. All designs were evaluated in wet labs.

Four dual-lumen SG concepts (I–IV) were designed to combine LV inflow and transvalvular OG into a single device (Fig. 1). All SG designs consisted of an inflow segment and a 12-mm OG measuring 14.6 cm in length, intended to cross the AV and terminate in the mid AAo. A circumferential polyester collar served as a sewing collar, enabling myocardial SG fixation at the LV apex.

The concepts differed in inflow geometry, diameter, and structural reinforcement. Concept I featured a crescent-shaped inflow with an outer diameter of 26 mm and transition to a circular cross-section. Concepts II–IV employed circular inflow geometries to facilitate connection to the LVAD. Additional modifications included variation in inflow length and the integration of metallic inlays to increase radial strength and maintain luminal patency under myocardial compression. Concept IV further incorporated a circumferential spring to reduce the risk of inflow collapse. The prototypes were manufactured by an external partner (Artivion, Inc., Kennesaw, GA, USA). For in vitro testing, a rigid 3D-printed model (Objet260 Connex, Stratasys Ltd., Eden Prairie, MN, USA) with identical dimensions was produced.

### In vitro testing

The SG model was evaluated in a mock circulatory loop using a centrifugal-flow LVAD (HM3). The loop consisted of two reservoirs filled with 2.5 L of a water–glycerol mixture (60/40% vol/vol), with a viscosity approximating that of blood at a hematocrit of 50%.

Pump speed was set to 4,000, 5,200, and 6,400 rpm, and afterload was adjusted using a diaphragm valve. Flow rates were measured downstream using a float-type flow meter (M335, SAFI, Taulignan, France). Pressures were recorded at the device inflow and outflow (RS PRO, RS Group plc, London, UK). Data acquisition was performed using a multi-channel analog-to-digital converter (RedLab 2416-4-AO, Meilhaus Electronic GmbH, Alling, Germany). Measurements were obtained under steady-state conditions and compared with previously acquired reference measurements from the identical setup without the SG model^13^. Two HM3 devices were tested to ensure consistency.

### Wet-lab study on explanted porcine heart

Wet-lab experiments were performed using explanted porcine hearts obtained from a local slaughterhouse. The implantation procedure was iteratively refined. Following deployment, SG expansion at the LV apex was assessed with and without balloon dilatation, and myocardial integrity was evaluated.

To quantify myocardial displacement independently of SG implantation, a non-compliant balloon catheter (Edwards Commander Delivery System, 29 mm diameter, Edwards Lifesciences, Irvine, CA, USA) was inserted into the LV apex of five explanted porcine hearts. Balloon inflation was performed in ten 10 mL increments up to a total balloon volume of 100 mL using a syringe pump (Twin Syringe Pump Model 33, HARVARD Apparatus, Holliston, MA, USA). Intraluminal balloon pressure was continuously recorded.

Balloon diameter and pressure were compared to a control condition without myocardial constraint. Myocardial tearing was assessed by external inspection and documented by photo and video analysis. Tear location was recorded on a 360° scale (0° anterior, 90° lateral, 180° posterior, 270° septal). Tear length and width were measured using a caliper.

### Animal testing

**Fig. 2:**
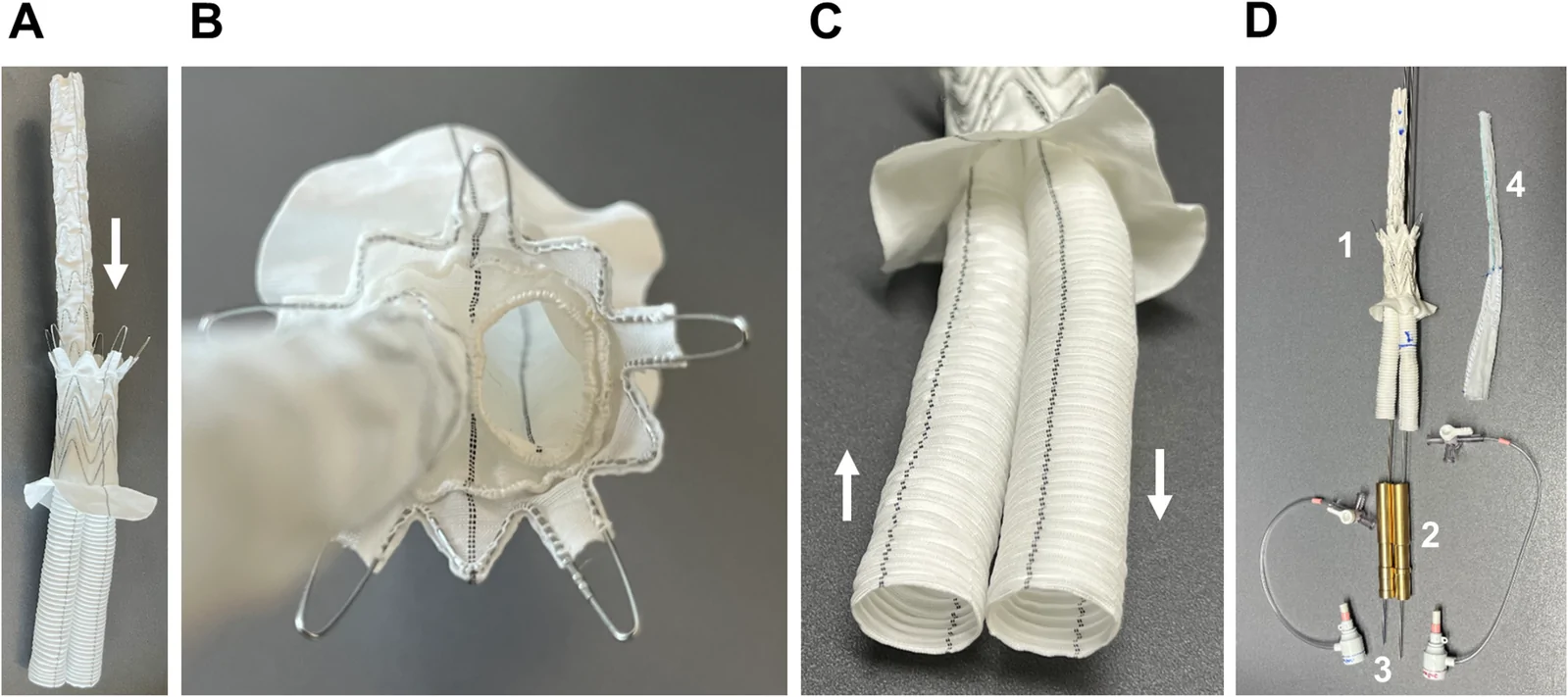
Stent graft Concept IV. (**A**) Side view showing inflow (arrow) and outflow (left). (**B**) Top view with inflow on the right. (**C**) Length-adjustable Dacron grafts for LVAD connection. (**D**) Components: (1) stent graft, (2) metallic inlays for LVAD connection, (3) introducer sheaths for blood sealing during guidewire advancement, and (4) sleeve for crimped stent graft insertion.

Based on the wet-lab findings, exploratory in vivo testing assessed transvalvular SG positioning under physiological beating-heart conditions and whether the identified limitations persisted despite structural SG reinforcement. The animal experiment aimed to evaluate the effects of myocardial contraction, intracardiac pressure, cyclic loading, and physiological MV and AV motion on SG expansion, apical sealing, and device stability. Concept IV was selected because its metallic inlays and circumferential reinforcement were intended to improve radial resistance against myocardial compression and maintain luminal patency (Fig. 2).

An acute feasibility study was performed in a healthy juvenile German domestic crossbred farm pig (German Landrace × Large White) weighing 107 kg, because of anatomical and physiological similarities to the human heart^14^. The study protocol was approved by the local animal welfare committee (Department of Agriculture, Rural Affairs, Veterinary and Food Services, Regierungspräsidium Freiburg, Germany, 35-9185.81/G-22/006) and conducted in accordance with the German Animal Protection Act, the European Convention for the Protection of Vertebrate Animals Used for Experimental and Other Scientific Purposes, and ARRIVE guidelines 2.0^15,16^.

A local farmer in the southwest of Baden-Württemberg, southern Germany, raised the animal under defined hygienic conditions. Permission was obtained from the farmer to use the pig in experiments. After the animal’s purchase and delivery to the University of Freiburg’s experimental animal facility, the animal’s clinical condition was assessed by experienced staff veterinarians. Special attention was paid to transport-related injuries, signs of diarrhea, breathing, and the animal’s behavior while standing still and moving. The skin was checked for signs of ectoparasites and infections.

The animal was housed under controlled environmental conditions (20°C, 75 ± 5% humidity, 13/11-hour light/dark cycle). It received standard pellet chow (20 g/kg) and water ad libitum. The animal underwent total intravenous anesthesia for surgery. For initial sedation, the it received pre-medication with a combination of ketamine (20 mg/kg IM) and midazolam (0.5 mg/kg IM). Anesthesia was induced using propofol (2–4 mg/kg IV) and vecuronium (0.2 mg/kg IV). Following endotracheal intubation, anesthesia and muscle relaxation were maintained with propofol (10–15 mg/kg/h IV), fentanyl (5–10 µg/kg/h IV), and vecuronium (0.2–0.4 mg/kg/h IV). Ringer’s solution (10 mL/kg/h IV) and noradrenaline (10–15 µg/kg/h IV) were administered to ensure fluid replacement and hemodynamic stability. Pressure-controlled ventilation was utilized and adjusted to maintain physiological conditions: FiO2 (20–40%), tidal volume (6–8 mL/kg), initial PEEP (5–8 mbar), and respiratory frequency (12–18/min). Continuous hemodynamic monitoring included arterial blood pressure, central venous pressure, and pulmonary artery pressure. Arterial blood samples were obtained at baseline, before LVAD activation, and before termination.

A median sternotomy was performed to maximize exposure and procedural control. Vascular access was obtained via the right jugular vein and right common carotid artery. A 24 F sheath was introduced for retrograde SG delivery. Stand-by CPB was prepared.

Epicardial and epiaortic echocardiography were used to guide guidewire passage, confirm SG positioning across the AV, assess MV and AV function, and detect intracardiac air^18^. After insertion into the right carotid artery, a guidewire was advanced across the AV into the LV and exteriorized through the LV apex. The delivery system was introduced retrogradely and exteriorized through the LV apex. Thereafter, it was loaded extracorporeally with the SG.

The system was retracted until the SG traversed the AV, then deployed and fixed at the apex via the circumferential SG collar. Additional SG expansion at the apex was attempted using a non-compliant balloon. The SG inflow and outflow were connected to a paracorporeal HM3. Pump support was initiated after de-airing.

The experiment was predefined as an exploratory proof-of-concept study. Termination criteria included uncontrollable bleeding, hemodynamic instability, or severe air embolism. The animal was euthanized with potassium chloride (2 mmol/kg IV). The explanted heart was examined for SG position, AV integrity, structural damage, and thrombus formation within the LV, the aortic root, the SG and the pump.

### Statistical analysis

Continuous variables are presented as mean ± standard deviation and compared using mean differences (MD) and two-sided Student’s t-tests where applicable. A p-value < 0.05 was considered statistically significant. Statistical analyses were performed using R (version 2021.9.0.351, RStudio, PBC, Boston, MA, USA). Data visualization was generated using the ggplot2 package (version 3.5.2)^19^.

### Use of artificial intelligence tools

During the preparation of this manuscript, ChatGPT (OpenAI) and Grammarly (Grammarly Inc.) were used to assist with language editing. All modified content was reviewed, verified, and approved by the authors, who take full responsibility for the final manuscript.

## Results

The SG concepts were evaluated in a staged preclinical workflow comprising in vitro, ex vivo, and exploratory proof-of-concept in vivo testing.

### In vitro performance

In vitro testing showed no statistically significant differences between the SG and control conditions. Compared to the control setup without SG, mean differences were 0.07 L/min for flow rate (*p* = 0.889) and 3.33 mmHg for pressure head (*p* = 0.764).

### Wet-lab evaluation of device expansion and myocardial interaction

**Fig. 3:**
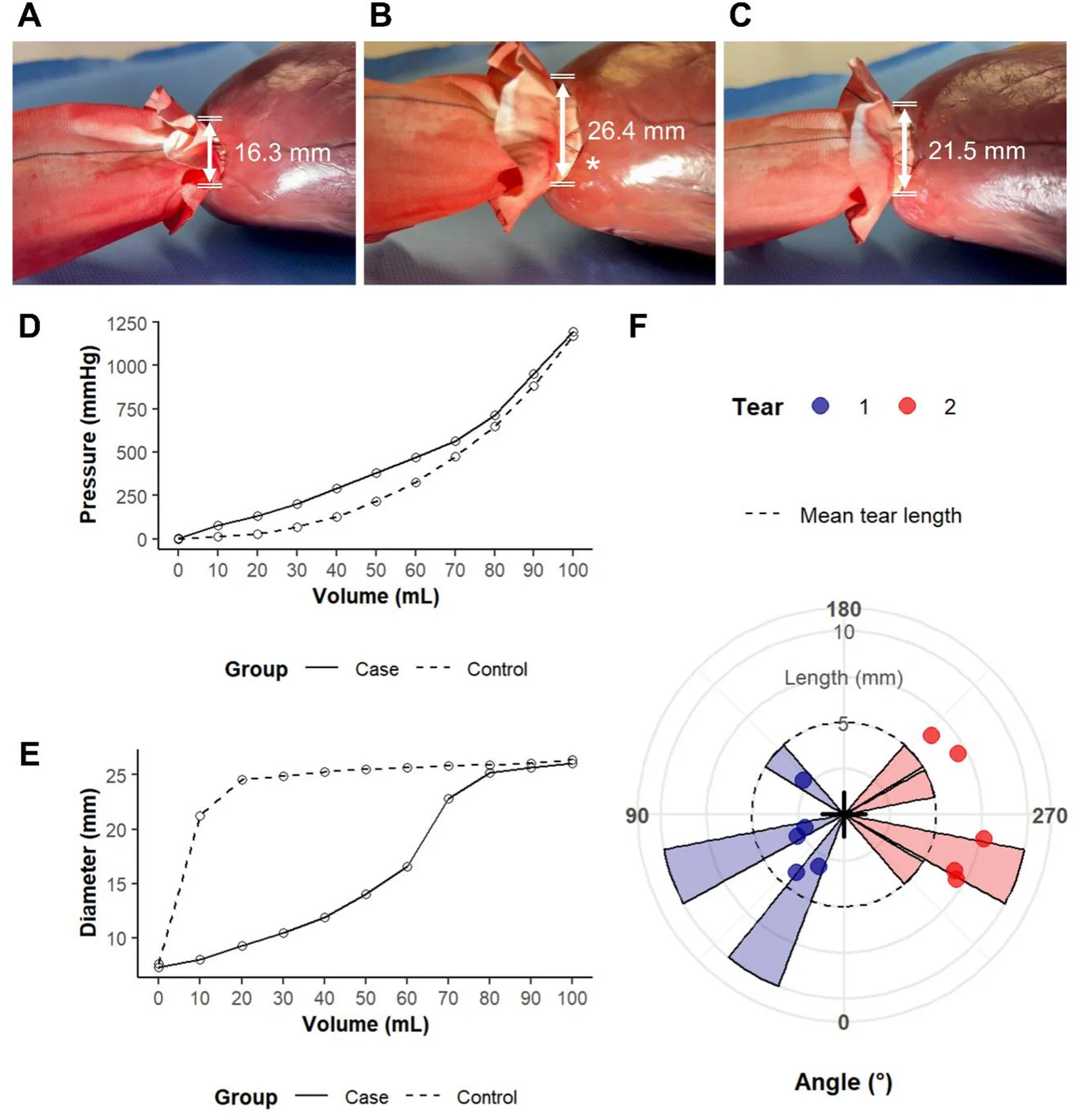
Stent graft behavior and myocardial displacement at the left ventricular apex. (**A**) Incomplete stent graft expansion. (**B**) Myocardial tearing (*) during balloon dilatation. (**C**) Compression by surrounding myocardium after balloon removal. (**D**) Pressure–volume curve for Case (with porcine heart) and Control (balloon only, without heart). (**E**) Diameter–volume relationship. (**F**) Myocardial tearing induced by balloon dilatation, showing tear location (angle) and length. Tear angle is depicted on a 360° scale (0° anterior, 90° lateral, 180° posterior, 270° septal).

Wet-lab testing confirmed that transapical SG implantation and transvalvular positioning were technically feasible. However, two reproducible failure modes were identified: incomplete SG expansion and myocardial tearing. In Concept I (outer diameter d_0_ = 26.0 mm), insufficient expansion resulted in luminal compression, reducing the effective diameter to d_1_ = 16.3 mm (Fig. 3A). Balloon dilatation restored expansion (d_2_ = 26.4 mm) but induced myocardial tearing (Fig. 3B). After balloon removal, recurrent myocardial compression reduced the diameter to d_3_ = 21.5 mm (Fig. 3C).

### Wet-lab quantification of myocardial displacement

Incremental balloon inflation in explanted porcine hearts (*n* = 5) resulted in an intraluminal pressure increase to 1,248 ± 107 mmHg (*p* < 0.001), accompanied by a balloon diameter increase from 7.3 ± 0.5 mm to 26.1 ± 0.3 mm (*p* < 0.001) (Fig. 3D, E).

Compared to control conditions without myocardial constraint (*n* = 1), pressure increased more steeply, while diameter expansion was delayed, indicating mechanical constraint imposed by the surrounding myocardial tissue. Myocardial tearing consistently occurred at balloon diameters exceeding 23 mm. Tears were observed at apicolateral (66 ± 40°) and apicoseptal (269 ± 33°) locations. Mean tear dimensions were 6.7 × 3.0 mm and 9.6 × 7.0 mm, respectively, with a median length of 5 mm (range 2–8 mm) (Fig. 3F).

### In vivo evaluation

**Fig. 4:**
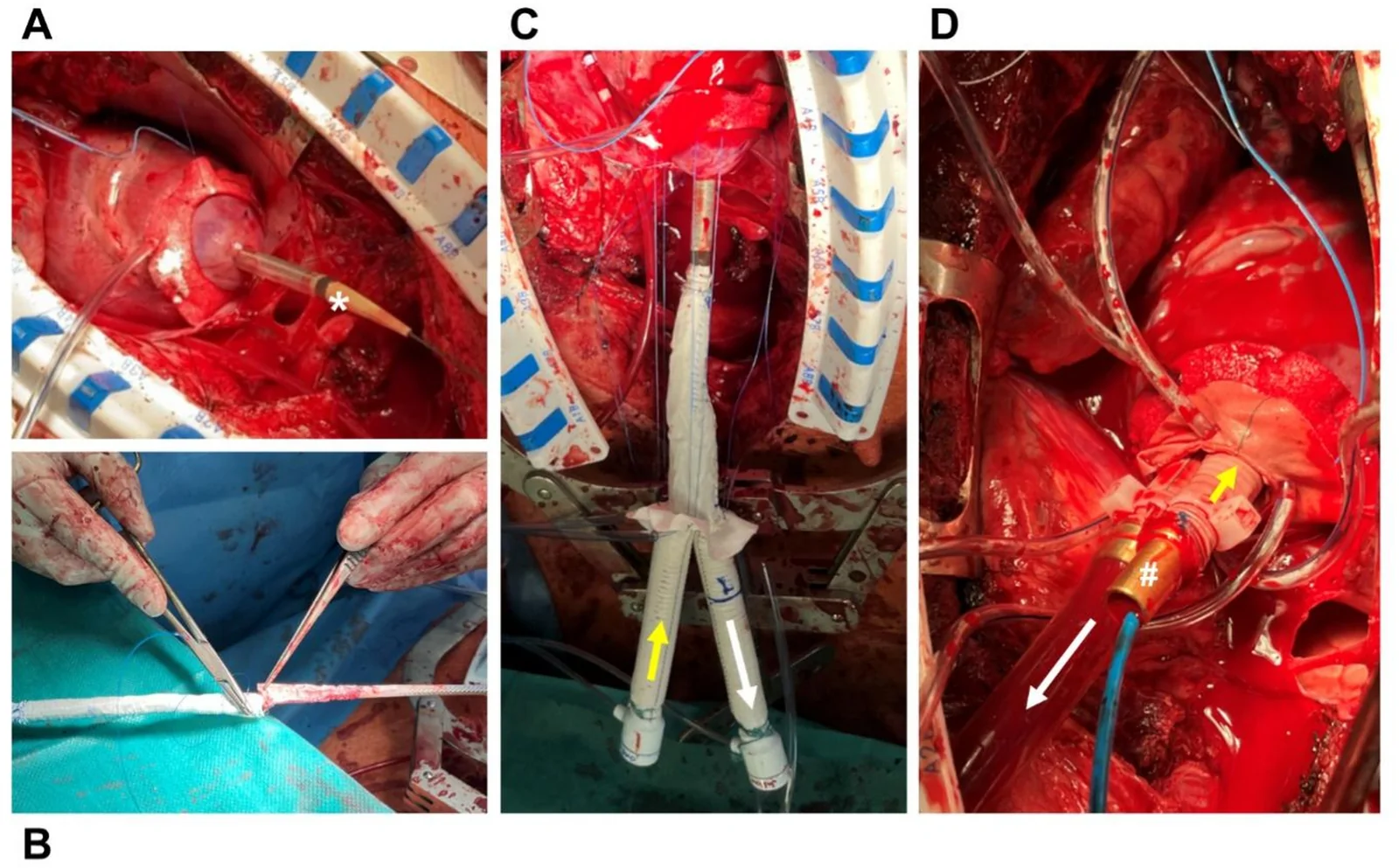
Implantation of the early-stage stent graft (SG) prototype. (**A**) Transapical exteriorization of the delivery system (*). (**B**) Suturing sleeves from the SG and the delivery system. (**C**) Partial retraction of the delivery system back through the left ventricle, on beating heart and without cardiopulmonary bypass, showing the extracardial inflow (white arrow) and outflow (yellow arrow). (**D**) Extracardial inflow and outflow after final SG positioning, and attachment to metallic inlays (#).

In vivo testing demonstrated successful transvalvular SG positioning but revealed procedural and physiological limitations. The reinforced SG (Concept IV) was positioned across the AV under echocardiographic guidance (Fig. 4), with no relevant AV regurgitation observed. Transapical delivery without LV coring was technically challenging and required radial incision of the apex to prevent invagination.

Under LVAD support (4,000 rpm), the estimated HM3 flow rate was 0.9 L/min, consistent with insufficient LV unloading. Mean arterial pressure decreased from 110 ± 12 mmHg at baseline to 34 ± 11 mmHg (*p* < 0.001). The experiment was terminated 11 min after LVAD activation due to air embolism and bleeding at the apical insertion site. Total procedure time was 5 h, with an estimated blood loss of 2 L. Hemoglobin decreased from 8.9 to 7.1 g/dL, accompanied by reductions in erythrocyte and platelet counts. Laboratory markers of hemolysis increased after LVAD activation, with the hemolysis index rising from 58 to 132 and plasma-free hemoglobin from 10 to 126 mg/dL.

### Postmortem findings

**Fig. 5:**
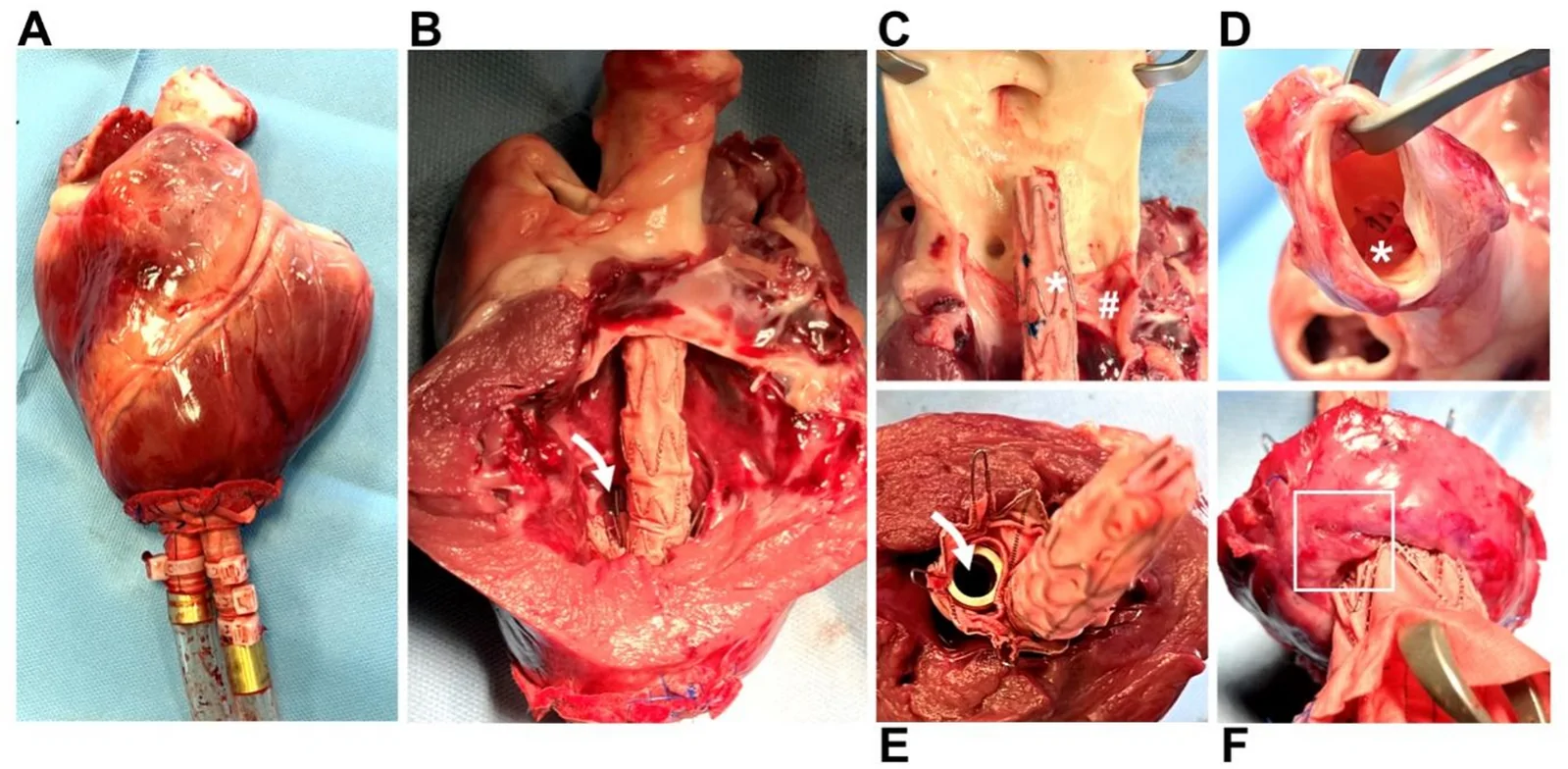
Postmortem inspection of the heart and left ventricle (LV). (**A**) Explanted heart with transapical stent graft connected to tubing. (**B**) LV chamber showing inflow (arrow) and outflow graft. (**C**) Distal portion of the outflow graft (*) crossing the aortic valve (#). (**D**) Outflow graft tip (*) positioned within the ascending aorta. (**E**) View from the LV into the inflow (arrow), supported by a metallic inlay. (**F**) External LV apex view after partial stent graft retraction, showing minor myocardial tearing (□).

Postmortem examination confirmed correct SG positioning (Fig. 5A, B), with the OG opening located in the mid AAo (Fig. 5C, D) and no macroscopic injury to the AV. The inflow lumen was compressed by the surrounding apical myocardium but remained partially patent due to the metallic inlay (Fig. 5E). A myocardial tear measuring 12 × 3 mm was identified at the apical insertion site (Fig. 5F).

## Discussion

This study systematically evaluated the hypothesis that a dual-lumen SG could be deployed transapically, with its OG crossing the AV, while maintaining sufficient LVAD performance and SG patency. Using a staged preclinical workflow, we identified three principal failure modes that limit the applicability of the current SG design: incomplete SG expansion, myocardial tearing at the apical insertion site, and insufficient apical sealing resulting in bleeding and air entry (Table 2).

**Table 2:** Failure modes, underlying causes and potential technical solutions.

Failure mode	Underlying cause	Potential technical solution
Incomplete SG expansion resulting in insufficient LV unloading	External compression of the inflow segment by the contracting LV myocardium at the apex, despite partial metallic reinforcement	Rigid or semi-rigid inflow cannula, direct pump–inflow integration, optimized intramyocardial geometry (e.g., semicircular shape of inflow)
Myocardial tearing at the apical insertion site	Mechanical mismatch between the radially expanding SG and the contracting myocardium, resulting in localized stress concentrations	Reduced insertion diameter, more controlled apical SG expansion, combination of partial apical coring and displacement to reduce stress
Insufficient apical sealing resulting in bleeding and air entry	Inadequate sealing between the SG and the contracting myocardium, aggravated by myocardial tearing	Dedicated apical sealing (e.g., cuff or gasket), additional SG coating, optimized apical access geometry, gentle myocardial approximation using pledgeted U-sutures

LV, left ventricle; SG, stent graft.

The ex vivo and in vivo results indicate that the primary limitation of the concept lies in SG–myocardium interaction at the transapical insertion site. Tissue displacement without coring creates a trade-off between apical SG expansion and myocardial injury. Unlike vascular structures, myocardial contraction exposes intraventricular devices to cyclic loading and repetitive geometric changes, which may compromise device stability and function^20^. These findings suggest an inherent biomechanical limitation of adapting conventional SG designs to the contracting myocardium. While increasing radial strength may improve SG patency, it also increases myocardial stress and the risk of tearing, whereas lower radial strength favors SG compression. The opposing constraints are difficult to overcome by incremental design improvements, suggesting that future concepts may require alternative designs and materials, potentially evolving from rigid cannula-based systems. Rigid concepts with trans- or subvalvular outflow have reached preclinical and, for selected systems, early clinical evaluation (Table 3). However, except for a dual-lumen cannula connected to an extracorporeal pump, all durable MCS systems require apical coring. To our knowledge, the present study is, the first to evaluate an expandable dual-lumen SG for transapical off-pump implantation based solely on myocardial displacement.

**Table 3:** Transapical MCS devices with transvalvular or subvalvular outflow.

Mechanical circulatory support	Extraventricular LVAD	Intraventricular LVAD	Intraventricular LVAD	Extracorporeal ECLS
Outflow	Transvalvular graft	Transvalvular cannula	Subvalvular outflow	Transvalvular cannula
Example	Herein described stent graft concept	tMVAD (HeartWare Inc., Framingham, MA, USA)^5,6,8^	ICOMS FlowMaker (FineHeart S.A., Bordeaux, France)	ProtekDuo (LivaNova, London, UK)
Pump	Combined with centrifugal pump	Integrated axial pump	Integrated axial pump	Extracorporeal roller pump
Support	Durable	Durable	Durable	Temporary
Flow path design	Side-by-side dual lumen	Axial single lumen	Axial single lumen	Concentric dual lumen
Apical access	Myocardial displacement	Apical coring	Apical coring	Myocardial displacement
Outflow diameter	12 mm	~ 12 mm	~ 8–10 mm	6.17 mm
Outflow material	Woven polyester graft^21^	Polymer cannula	Titanium	Polyurethane cannula
Development stage	Preclinical	Preclinical^5,6^	Early clinical^23^	Clinical^24^

ECLS, extracorporeal life support; ICOMS, Implantable Cardiac Output Management System; LVAD, left ventricular assist device; tMVAD, transapical miniaturized ventricular assist device.

Miniaturized intraventricular axial-flow pumps aim to reduce invasiveness, bridging the gap between temporary MCS devices and durable LVADs^5,6,8^. Related concepts combine miniaturized extraventricular centrifugal pumps positioned at the LV apex with a short, rigid inflow cannula and a transventricular outflow cannula arranged side by side. Without an intermediate connector, as required for the herein proposed SG, the mechanical lever arm between pump head and apical fixation site is reduced. Rigid materials may additionally lower the risk of kinking and external compression. Rotating the pump head by 90° relative to its current position could allow direct alignment of the pump outflow with the transventricular outflow cannula. A low-profile pump head may reduce apical LV compression and facilitate implantation in smaller patients.

So far, the long-term effects of transvalvular outflows on the AV are unknown. Although a 30-day large-animal study investigating a solid transvalvular LVAD outflow cannula revealed preserved cusp coaptation without structural damage^5^, longer-term studies are required to characterize chronic device–AV interaction and valve function. Until these effects are better understood, such concepts may be best suited for intermediate-term MCS. AV-related effects are expected to depend not only on MCS duration but on central positioning and alignment of the OG or outflow cannula.

While the present OG concept inherently provides self-centering within the AV, which is favored by its dynamic geometry, rigid cannulas may require dedicated centering mechanisms, such as anchoring struts^25^ or expandable stabilizing elements. In cases requiring AV replacement, centering with an additional AV stent^26^ or a stent-anchored AV, in which the non-coronary cusp is occupied by the OG^25^, may represent an alternative strategy.

In case of myocardial recovery, established LVAD weaning protocols would require adaptation, as the transvalvular OG may interfere with AV function. Assessment should combine gradual reduction of MCS with echocardiographic evaluation of LV dimensions, LV function, and AV opening and competence. After prolonged support, thrombotic or fibrotic changes may impair valve function after device removal, potentially resulting in relevant AR, LV volume overload, and compromised myocardial recovery, with AV repair or replacement required in severe cases.

For a 12-mm OG, reported aortic sinus diameters of 26.2 ± 2.3 mm in German Landrace pigs^27^ and 36.7 ± 4.6 mm in LVAD patients^10^ correspond to theoretical radial clearances of approximately 7.1 and 12.4 mm, respectively, assuming central positioning. Eccentric alignment or smaller aortic roots may reduce local clearance and potentially compromise coronary perfusion. In addition, impaired aortic root washout may promote local stasis and thrombus formation, leading to secondary coronary ostial obstruction.

Beyond aortic and valvular concerns, transventricular outflows also alter intraventricular flow patterns^12^. They may impair LV washout and create low-flow regions between the cannula and the LV wall, potentially promoting LV wall thrombosis. Previous in vitro and in silico studies have reported diverging effects of inflow cannula length on LV washout, apical stasis, and thrombogenicity^12,28,29^.

Less-invasive beating-heart LVAD implantation through lateral thoracotomy represents an established alternative to median sternotomy. The present concept was designed to avoid an aortic OG anastomosis through a single transapical access. However, transventricular manipulation of the beating LV introduces risks of air embolism, bleeding, limited intracardiac visualization, and potential emergency conversion to CPB. While CPB-assisted implantation could mitigate some of these risks, avoiding CPB remained a primary design objective to reduce procedural complexity and avoid CPB-related complications. Thus, successful transapical off-pump MCS requires dedicated strategies for transapical sealing and prevention of air entry without relying on routine CPB support.

This exploratory proof-of-concept study has several limitations. The in vivo evaluation was limited to a single acute experiment. It was intended primarily for failure-mode characterization rather than statistical assessment of feasibility. The surgical approach involved median sternotomy and paracorporeal LVAD support, which do not reflect the intended clinical application. In addition, the porcine model differs from the clinical target population, particularly in LV geometry and myocardial thickness^14^. Finally, echocardiographic data could not be stored due to a technical failure.

In general, early identification of failure modes is critical for cardiovascular device development. Whenever possible, critical failure modes should be identified and addressed in in vitro and ex vivo models before proceeding to in vivo evaluation, thereby improving development efficiency and reducing unnecessary animal use. In this context, transparent reporting of unsuccessful approaches may reduce publication bias and support the generation of balanced datasets for data-driven methodologies^30^.

In conclusion, despite preserved in vitro performance, the novel dual-lumen SG for transapical LVAD implantation is limited by myocardial compression and insufficient apical sealing, which can lead to bleeding and air embolism. The failure modes observed across different experimental models highlight the importance of resolving critical device–tissue interactions before further in vivo evaluation and may inform future design strategies for transapical MCS systems deployed without apical coring.

## Data Availability

The datasets used and analyzed during the current study are available from the corresponding author upon reasonable request.
